# Raising the Standard of the Medical Education—The Way to Do It

**Published:** 1890-03

**Authors:** 


### Editorial Department.

F. E. DANIEL, M. D., EDITOR AND PUBLISHER.

THIS JOURNAL, by unanimous vote of the members, was made the Official Organ of the Austin District Medical Society.

COLLABORATORS:

*Dr. R. M. Swearingen, Austin.*
*Prof. B. E. Hadra, M. D., Galveston.*
*Prof. Geo. Cupples, M. D., San Antonio.*
*Dr. T. C. Osborn, Cleburne, Texas.*
*Dr E. J. Doering, Chicago.*
*Dr. E. J. Beall, Fort Worth.*
*Dr Odo Betz, Germany.*
*Dr. J. W. McLaughlin, Austin.*
*Dr. T. J. Tyner, Austin.*
*Prof. J. F. Y. Paine, M. D., Galveston.*
*Dr. R. H L. Bibb, Mexico.*
*Dr. T. J. Bennett. Austin.*
*Dr. Bat Smith, Wharton.*
*Dr. E. Meierhof, New York.*
*Prof. W. B. Rogers, M. D., Memphis, Tenn.*

### RAISING THE STANDARD OF MEDICAL EDUCATION—THE WAY TO DO IT.

The necessity for better educated Doctors is universally felt. Several attempts have been made to devise a plan whereby the standard of instruction could be fixed and adhered to; and the one idea has been—co-operation; but the trouble is—that, in order to secure larger, and consquently, better paying classes, certain colleges have insisted on cheaper rates and easier graduation; and these have been held out as inducements to men whose preliminary education—or, rather, we should say—whose lack of preliminary education should have prevented their ever trying to become Doctors. Some years ago, it will be remembered, a large number of medical colleges formed an association, and agreed to require a three years graded course, with preliminary examination. In theory, this was a good move, but, after one year's trial, certain large colleges found that their classes (and consequently their receipts) fell off; and, disregarding their compact with the other colleges, they went back immediately, to the old plan. We call no names; they are known. It was a case of the dog returning to his vomit, and—the *status quo* obtains to-day, we believe.

Recently, two Eastern colleges, finding themselves left as to numbers—have issued a circular, requesting a meeting of college representatives at Nashville, in May, during the session of the American Medical Association, with a view of securing the co-operation of a majority in an advance ; an agreement that each one shall require a higher standard, and fix the curriculum. Well, it will fail, as before, and for the same reason ; there are too many colleges run for money making, principally, and a strict examination and a thorough and protracted course of study will drive off the class above referred to. Strange, no one seems to have thought of setting an example, of acting *solus*, of putting his shoulder to the wheel and then calling for help and Hercules.

Now, in our judgment, the way to accomplish the desired result is, that each college whose ambition is to teach medicine and earn (or maintain) a high character shall act independently of all others, and regulate their sessions and curriculum with a view of imparting a thorough medical education, without reference to what others may do ; and they will find, after awhile—for the leaven of reform is working—that it will pay. The graduates of cheap colleges will, in the course of time, be a distinct class ; and when that glorious day shall dawn, when there will be examining boards in all the States, these will be compelled to complete their education or drop out ; the sifting process will let through only those who left college prepared to stand an examination.

Without intending to be invidious, we cannot resist the inclination to indicate, in this connection, the Medical Department of the University of Tennessee ; because the faculty of that school have inaugurated a move, and are carrying out precisely the JOURNAL'S idea as given above. Independently of any other they have determined to make education at their college as thorough as money, facilities, ability and time can make it. Dr. W. J. Matthews of this city, a practitioner of fifteen years, attended the session just closed and took the degree. He informs us that the new museum equipment of that school now being received from Paris, embraces all the new and improved models and designs for teaching anatomy, physiology and obstetrics ; amongst which are a splendid copy of Anzeaux' clastic or dissected model of man (which alone cost $750), a model of the female in wax, true to life, and illustrating every feature in obstetrics; models of the arterial, venous and lymphatic systems; sets of models showing the pathology and progress of all kinds of skin diseases, etc.; syphilis, cancer, tumors, etc.; uterine displacements, hernias, etc., etc. They have, also, for teaching comparative anatomy, a complete set of busts of nationalities. By next session, a library and reading room will be added, where all the home and foreign medical journals and all recent medical publications will be kept for the use of the students. Microscopy will be thoroughly taught; and to this end the college has supplied a large lot of expensive instruments, with histological and pathological specimens; chemestry too, will be taught practically, and the apparatus and re-agents, for the use of the class in chemistry, is as complete as money and skill can make them; electricity in its application to medical practice, will be taught in all its phases, and the electrical apparatus is on the same extensive scale as the other equipments. Outside of the regular course, instruction will be given in these branches to those who desire to make a specialty of either; but instruction in each will be embraced in the regular curriculum; and every device and facility will be afforded the classes for acquiring knowledge in accordance with the most advanced ideas of the day; hence students turned loose from the University of Tennessee hereafter, and armed with their diploma, will enter the arena of practice with a large advantage over those taught under the old system; they will be, moreover, familiar with modern authors, and up with the most advanced theories and tenets. The faculty of this college embraces two Professors of surgery and two of medicine, besides a full corps of teachers in all the special branches, and taken, all together, is one of the largest and most complete of any medical school in the South.

These gentlemen have not stopped to ask their competitors, "what are you going to do?" nor whether it will pay, but with a laudable desire to be foremost in the good work of raising the standard of medical education in the South, they have gone ahead and spent immense amounts of money, in order that they may be prepared to teach medicine as it is to-day understood and practiced in the great medical centers of the world. Let other colleges speedily follow their example and in a short while the problem will be solved and there will be no more complaint of half educated Doctors turned loose upon the people.

